# Supplementary material for: Resolving the full spectrum of human genome variation using Linked-Reads
Source: Genome Res. 2019 Apr;29(4):635–45. doi: 10.1101/gr.234443.118 (PMC6442396; doi:10.1101/gr.234443.118)
Supplement: Supplemental Material [file supp_gr.234443.118_Supplemental_longranger-2.2.zip › longranger-2.2.tar.bz2!/lr2.2-public/lib/stan/cmdstan/stan/doc/api/html/check__not__nan_8hpp_source.html]

Stan: src/stan/math/error\_handling/check\_not\_nan.hpp Source File


|  |  |
| --- | --- |
|  | Stan  2.5.0  probability, sampling & optimization |


- Main Page
- Related Pages
- Namespaces
- Classes
- Files

- File List
- File Members

All Classes Namespaces Files Functions Variables Typedefs Enumerations Enumerator Friends Macros Pages

- src
- stan
- math
- error\_handling

check\_not\_nan.hpp

Go to the documentation of this file.

1 #ifndef STAN\_\_MATH\_\_ERROR\_HANDLING\_CHECK\_NOT\_NAN\_HPP

2 #define STAN\_\_MATH\_\_ERROR\_HANDLING\_CHECK\_NOT\_NAN\_HPP

3

4 #include <stan/math/error\_handling/dom\_err.hpp>

5 #include <stan/math/error\_handling/dom\_err\_vec.hpp>

6 #include <boost/math/special\_functions/fpclassify.hpp>

7 #include <stan/meta/traits.hpp>

8

9 namespace stan {

10  namespace math {

11

12  namespace {

13  template <typename T\_y, typename T\_result, bool is\_vec>

14  struct not\_nan {

15  static bool check(const char\* function,

16  const T\_y& y,

17  const char\* name,

18  T\_result\* result) {

19  if ((boost::math::isnan)(y))

20  return dom\_err(function,y,name,

21  " is %1%, but must not be nan!","",

22  result);

23  return true;

24  }

25  };

26

27  template <typename T\_y, typename T\_result>

28  struct not\_nan<T\_y, T\_result, true> {

29  static bool check(const char\* function,

30  const T\_y& y,

31  const char\* name,

32  T\_result\* result) {

33  // using stan::length;

34  for (size\_t n = 0; n < stan::length(y); n++) {

35  if ((boost::math::isnan)(stan::get(y,n)))

36  return dom\_err\_vec(n,function,y,name,

37  " is %1%, but must not be nan!","",

38  result);

39  }

40  return true;

41  }

42  };

43  }

44

55  template <typename T\_y, typename T\_result>

56  inline bool check\_not\_nan(const char\* function,

57  const T\_y& y,

58  const char\* name,

59  T\_result\* result) {

60  return not\_nan<T\_y,T\_result,is\_vector\_like<T\_y>::value>

61  ::check(function, y, name, result);

62  }

63

64  }

65 }

66 #endif

boost::math::isnan

bool isnan(const stan::agrad::var &v)

Checks if the given number is NaN.

**Definition:** boost\_fpclassify.hpp:69

dom\_err.hpp

stan::math::dom\_err

bool dom\_err(const char \*function, const T &y, const char \*name, const char \*error\_msg, const T\_msg error\_msg2, T\_result \*result)

**Definition:** dom\_err.hpp:36

stan::length

size\_t length(const T &)

**Definition:** traits.hpp:159

stan::math::dom\_err\_vec

bool dom\_err\_vec(const size\_t i, const char \*function, const T &y, const char \*name, const char \*error\_msg, const T\_msg error\_msg2, T\_result \*result)

**Definition:** dom\_err\_vec.hpp:33

traits.hpp

stan::math::check\_not\_nan

bool check\_not\_nan(const char \*function, const T\_y &y, const char \*name, T\_result \*result)

Checks if the variable y is nan.

**Definition:** check\_not\_nan.hpp:56

dom\_err\_vec.hpp

stan::get

T get(const T &x, size\_t n)

**Definition:** traits.hpp:143


---

[ Stan Home Page ]

*© 2011–2014,
Stan Development Team.*
